# Analysis and Prediction of Corrosion of Refractory Materials by Sodium Salts during Waste Liquid Incineration—Thermodynamic Study

**DOI:** 10.3390/ma13214729

**Published:** 2020-10-23

**Authors:** Guishi Cheng, Ying Zhao, Fei Long, Jiahe Zhang, Tengfei Zhao, Lu Liu, Xiaoqiang Wang, Changqing Dong

**Affiliations:** 1 National Engineering Laboratory for Biomass Power Generation Equipment, School of New Energy, North China Electric Power University, Beijing 102206, China; 51101890@ncepu.edu.cn (G.C.); 120181100109@ncepu.edu.cn (J.Z.); zhao_012345@163.com (T.Z.); 51102247@ncepu.edu.cn (L.L.); energy@ncepu.edu.cn (X.W.); cqdong@ncepu.edu.cn (C.D.); 2 Department of Mechanical and Material Engineering, Queen’s University, Kingston, ON K7L 3N6, Canada; long.fei@queensu.ca; 3 State Key Laboratory of Alternate Electrical Power System with Renewable Energy Sources, North China Electric Power University, Beijing 102206, China

**Keywords:** sodium salt, refractory, corrosion, thermodynamics

## Abstract

Incineration of high-content sodium salt organic waste liquid will corrode the refractory material in the incinerator, causing the refractory material to peel off and be damaged. A thermodynamics method was used to study the thermodynamic properties of three common sodium salts (NaCl, Na_2_CO_3_ and Na_2_SO_4_) on the corrosion of refractory materials (MgO·Cr_2_O_3_, MgO·Al_2_O_3_, Al_2_O_3_, MgO and Cr_2_O_3_). The results determined that MgO has the best corrosion resistance and is not corroded by the three sodium salts. On this basis, the thermodynamic corrosion experiments of NaCl corrosion of magnesium oxide at three temperatures of 600, 1000 and 1200 °C were carried out. Analysis of the corrosion product by X-ray diffraction (XRD) showed no corrosion product formation. Studies have shown that thermodynamic calculation can accurately predict the thermodynamic mechanism of alkali metal corrosion to refractory materials, and MgO is a good anti-alkali metal corrosion refractory material.

## 1. Introduction

The rapid development of the petrochemical industry has led to an increase in emissions of high-concentration organic waste liquids. This type of wastewater is not only difficult to be decomposed by microorganisms, but also has the potential to induce genetic mutations and toxicity to humans and animals. Incineration is a commonly used method for treating organic waste liquids in industry, especially for some waste liquids with high concentration, complex composition and high heat value. However, the organic waste liquid contains alkali metal salts such as Na_2_SO_4_, KCl, NaCl, etc., and the acidic waste water is usually neutralized by adding alkaline substances (such as KOH, NaOH) before incineration to reduce the corrosion of the pump and the pipeline by the acidic wastewater, bringing more alkali metal salts to the organic waste liquid. Most alkali metal salts have a low melting point, causing severe corrosion to the refractory layer of the incinerator, affecting the service life of the refractory, and causing great safety hazards and economic losses to the operation of the incinerator [1].

With the advancement of society and the development of science and technology, the performance requirements of refractory materials are getting higher and higher. Many researchers have studied and improved the corrosion resistance of refractories [2,3,4,5,6], thermal shock resistance [7,8,9], and anti-wear properties [10]. M. Yoshikawa et al. [11] studied the erosion resistance of MgO-based spinel and Al_2_O_3_-based spinel refractories and found that these two refractories can replace Al_2_O_3_-Cr_2_O_3_ refractories with low chromium content and are more suitable for alkali furnaces. P. Prigent et al. [1] studied the sodium corrosion resistance of three types of silica-alumina refractories (andalusite, mullite, and refractory clay) and compared the microstructure of the refractory surface before and after NaF vapour corrosion. J. Moda et al. [12] added MgO and NiO to an alumina refractory to realize Al_2_O_3_-MgO-NiO refractories and found that a (Mg, Ni)O solid solution was formed in the material and that its ability to resist slag erosion was improved. R. D. Fan et al. [13] found that alumina chrome slag can replace alumina to prepare Al_2_O_3_-SiC-C troughe castables, which remarkably improved oxidation resistance of the sample, but reduced its thermo-mechanical properties and anti-slag corrosion performance. Some researchers have paid attention to the effect of additives on the corrosion resistance of refractory materials. K. Igabo et al. [14] added ZrO_2_ to MgO-Al_2_O_3_ refractories and studied the effect of the addition on the performance of the refractories. P. Gehre et al. [15] reported that adding spinel materials, spinel-rich cements, and calcium aluminate cement to the spinel-containing alumina lining of steel ladles could significantly improve the slag corrosion resistance.

There are many studies concerning the performance of refractories used in the steelmaking process [16,17,18,19], but the thermodynamic research on sodium salt corrosion of refractory materials is limited. D. Gregurek et al. [20] used Factsage software to carry out thermodynamic simulation on the corrosion of refractory lining caused by slag and CuO. The research results can provide a theoretical basis for the wear of refractories in copper anode furnaces. J. Stjernberg et al. [21] used XRD, electron microscopy and spectroscopy (Quantitative Evaluation of Minerals by Scanning Electron Microscopy (QEMSCAN) and Scanning Electron Microscopy (SEM)) characterization and thermodynamic kinetics to study the erosion of mullite based refractory bricks by alkali metals. R. Huang et al. [22] calculated the erosion of four refractory materials (i.e., magnesia carbon brick, burned magnesite brick, SiC castable, and corundum castable) by titanium slag using FactSage software. The results found that silicon carbide castable has the best corrosion resistance among the four refractories, because it can interact with Ti slag to form TiC with a high melting point, which can prevent Ti slag from penetrating more deeply into refractories.

Based on the thermodynamic study of refractory corrosion, this paper compares the corrosion resistance of five refractories under different sodium salts, providing a theoretical basis and data support for further research on alkali metal corrosion resistance of refractories.

## 2. Thermodynamic Calculations

Thermodynamic simulation is based on the version 7.2 of FactSage^TM^ software for thermodynamic equilibrium calculations. In this paper, the components of refractory materials such as MgO·Cr_2_O_3_, MgO·Al_2_O_3_, Al_2_O_3_, MgO and Cr_2_O_3_ are selected as research objects. The corrosion mechanism of Na_2_CO_3_, Na_2_SO_4_ and NaCl on refractory materials at different temperatures (600 °C–1200 °C with a temperature step of 100 °C) was investigated. The refractory materials components of MgO·Cr_2_O_3_, MgO·Al_2_O_3_, Al_2_O_3_, MgO and Cr_2_O_3_ and 3-sodium salts of 1mol were input in the table of the FactSage^TM^ software for the calculation. In this paper, the Equilib module of FactSage^TM^ 7.2 software was used, which is suitable for calculating the concentration of various species when the reaction of a given element or compound reaches chemical equilibrium.

## 3. Thermodynamic Experimental Procedure

**Figure 1:**
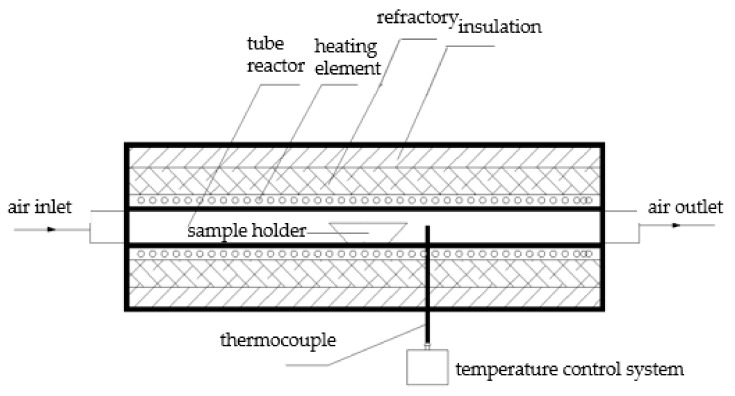
Schematic diagram of experiment setup.

Similar to our previous work [23], the experiment was conducted in a high-temperature tube furnace (Figure 1). A 1:1 molar ratio of NaCl and MgO was put into a corundum boat and full mechanical mixing was carried out. The rest of experimental and data analysis procedures were all the same as previously reported [23].

## 4. Results and Discussion

### 4.1. Corrosion Effect of Na_2_CO_3_ on Refractory Materials

**Figure 2:**
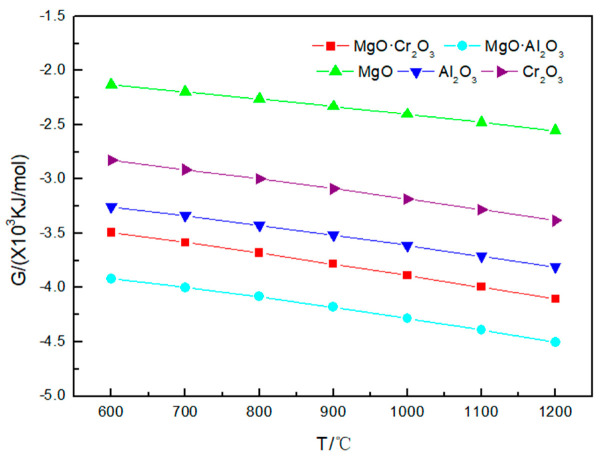
Gibbs free energy of the five corrosion reactions between Na_2_CO_3_ and refractory materials at 600–1200 °C.

Figure 2 illustrates that the Gibbs free energy values for the reactions of Na_2_CO_3_ with five refractory materials are all negative. As the temperature increases, the Gibbs free energy value decreases, indicating that with the increase in temperature, the corrosion tendency of refractories by Na_2_CO_3_ becomes more obvious. It can also be obtained from the figure that the order of the tendency of the five refractories to be corroded by Na_2_CO_3_ is MgO·Al_2_O_3_ > MgO·Cr_2_O_3_ > Al_2_O_3_ > Cr_2_O_3_ > MgO.

In general, MgO-based refractory materials are basically free from corrosion reaction in the temperature range below 1200 °C, because MgO ions carry two charges and the radii of oxygen ions and magnesium ions are relatively small, which causes magnesium oxide to have large lattice energy, a high melting point and stable properties [24].

The corrosion products of refractory materials corroded by sodium carbonate and the corrosion reaction equations deduced therefrom are shown in Table 1 shows that, in addition to MgO, other refractories are corroded by sodium carbonate at 600 °C.

The reaction products and reaction degree of Al_2_O_3_ and MgO·Al_2_O_3_ corroded by Na_2_CO_3_ are related to temperature. Al_2_O_3_ is more easily corroded by Na_2_CO_3_, and has been completely corroded at 600 °C to generate 0.16667 mol Na_2_Al_12_O_19_, and 2 mol of NaAlO_2_ at 700–1200 °C. At 600–1100 °C, only a small amount of MgO·Al_2_O_3_ is corroded to form NaAlO_2_ and MgO; when the temperature reaches 1200 °C, MgO·Al_2_O_3_ is completely corroded. The reaction of Al_2_O_3_ and MgO·Al_2_O_3_ by Na_2_CO_3_ corrosion is as follows:

Na_2_CO_3_ + 6Al_2_O_3_ = Na_2_Al_12_O_19_ + CO_2_ (1)

Na_2_CO_3_ + Al_2_O_3_ = 2NaAlO_2_ + CO_2_ (2)

Na_2_CO_3_ + MgO·Al_2_O_3_ = 2NaAlO_2_ +MgO + CO_2_ (3)

In the reaction of Cr_2_O_3_ and MgO·Cr_2_O_3_ by Na_2_CO_3_, Cr^3+^ is oxidized to Cr^6+^ due to the presence of O_2_, and the corrosion of Cr_2_O_3_ and MgO·Cr_2_O_3_ at 600–1200 °C by Na_2_CO_3_ is:

Na_2_CO_3_ + 1/2 Cr_2_O_3_ + 3/4 O_2_ = Na_2_CrO_4_ +CO_2_ (4)

Na_2_CO_3_ + 1/2 MgO·Cr_2_O_3_ + 3/4 O_2_ =1/2 MgO + Na_2_CrO_4_ + CO_2_ (5)

In addition, it can be seen from the table that no corrosion product of Na_2_CO_3_ corroding MgO is found. However, Na_2_CO_3_ will decompose at 600–1200 °C, so the Gibbs free energy of the MgO-Na_2_CO_3_ reaction is negative. To sum up, MgO is hard to be eroded by Na_2_CO_3_.

### 4.2. Corrosion Reaction of Na_2_SO_4_ and Refractory Materials

**Figure 3:**
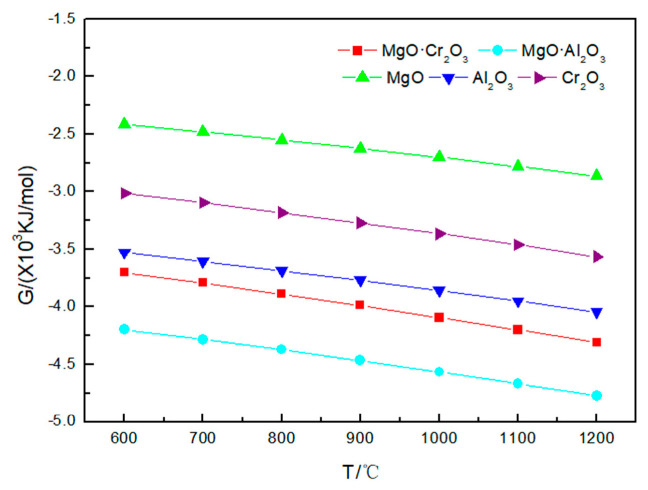
Gibbs free energy of the five corrosion reactions between Na_2_SO_4_ and refractory materials at 600–1200 °C.

As shown in Figure 3 and Table 2, under 600–1200 °C, neither MgO nor MgO·Al_2_O_3_ will be corroded by sodium sulfate. A small amount of Al_2_O_3_ (0.00135 mol) and Cr_2_O_3_ (0.0001 mol) will be corroded by sodium sulfate at 1000 °C. As the temperature rises to 1200 °C, more Al_2_O_3_ (0.1425 mol) and Cr_2_O_3_ (0.00365 mol) will be corroded by sodium sulfate. The reaction of Al_2_O_3_ and Cr_2_O_3_ corroded by sodium sulfate is:

Na_2_SO_4_ + 9Al_2_O_3_ = 2NaAl_9_O_14_ + SO_3_ (6)

Na_2_SO_4_ + 1/2 Cr_2_O_3_ + 3/4 O_2_ = Na_2_CrO_4_ + SO_3_ (7)

Similarly, a very small amount of MgO·Cr_2_O_3_ (0.00005 mol) will be corroded by sodium sulfate to form Na_2_CrO_4_ at 1100 °C With the temperature rises, even if the temperature rises to 1200 °C, as long as 0.0004 mol of MgO·Cr_2_O_3_ is corroded by sodium sulfate to form Na_2_CrO_4_, the reaction of MgO·Cr_2_O_3_ being corroded by sodium sulfate is:

Na_2_SO_4_ + 1/2 MgO·Cr_2_O_3_ + 3/4O_2_ = Na_2_CrO_4_ + 1/2MgO + SO_3_ (8)

From the above results, it can be seen that the order of corrosion degree of five refractories by Na_2_SO_4_ from strong to weak is as follows: Cr_2_O_3_ and Al_2_O_3_ > MgO·Cr_2_O_3_ > MgO·Al_2_O_3_ and MgO.

### 4.3. Corrosion Reaction of NaCl and Refractory Materials

**Figure 4:**
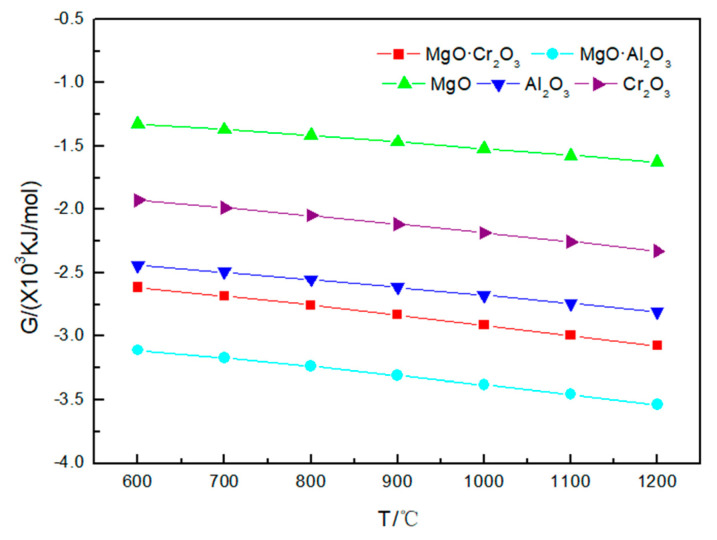
Gibbs free energy of the five corrosion reactions between NaCl and refractory materials at 600–1200 °C.

The results in Figure 4 show that the Gibbs free energy of the refractory material corroded by sodium chloride has a tendency to change with that of the refractory material with sodium carbonate and sodium sulfate at 600–1200 °C. I t can be seen from Table 3 that NaCl is less corrosive to refractory materials. Even at 1200 °C, MgO and MgO·Al_2_O_3_ are not corroded by sodium chloride; only a small amount of Al_2_O_3_ (0.00855 mol), Cr_2_O_3_ (0.0003 mol) and MgO·Cr_2_O_3_ (0.000005 mol) is corroded by sodium chloride, and the reaction equation is:

2NaCl + 9Al_2_O_3_ + 1/2 O_2_ = 2NaAl_9_O_14_ + Cl_2_ (9)

4NaCl + Cr_2_O_3_ + 5/2 O_2_ = 2Na_2_CrO_4_ + 2Cl_2_ (10)

4NaCl + MgO·Cr_2_O_3_ + 5/2 O_2_ = 2Na_2_CrO_4_ + MgO +2Cl_2_ (11)

It is worth noting that Figure 2, Figure 3 and Figure 4 shows that the Gibbs free energy of the reaction of MgO·Cr_2_O_3_ and MgO·Al_2_O_3_ corroded by sodium carbonate, sodium sulfate and sodium chloride is smaller than that of Al_2_O_3_ and Cr_2_O_3_ corroded by sodium carbonate, sodium sulfate and sodium chloride, but, as can be seen from Table 1, Table 2 and Table 3, MgO·Cr_2_O_3_ and MgO·Al_2_O_3_ are less susceptible to corrosion by sodium carbonate, sodium sulfate and sodium chloride than Al_2_O_3_ and Cr_2_O_3_. This is because MgO·Cr_2_O_3_ and MgO·Al_2_O_3_ will undergo decomposition reaction at high temperature to generate MgO and Cr_2_O_3_, MgO and Al_2_O_3_, respectively. This also shows that the trend of corrosion resistance of refractory materials cannot be judged solely by the Gibbs free energy of the reaction, but must be judged comprehensively by combining reaction products.

Therefore, Al_2_O_3_ and Cr_2_O_3_ are most easily corroded by sodium carbonate, sodium sulfate and sodium chloride, followed by MgO·Cr_2_O_3_, and MgO·Al_2_O_3_ and MgO, which are the most difficult to be corroded by sodium salts.

### 4.4. Thermodynamic Experiment of NaCl Corrosion of MgO

**Figure 5:**
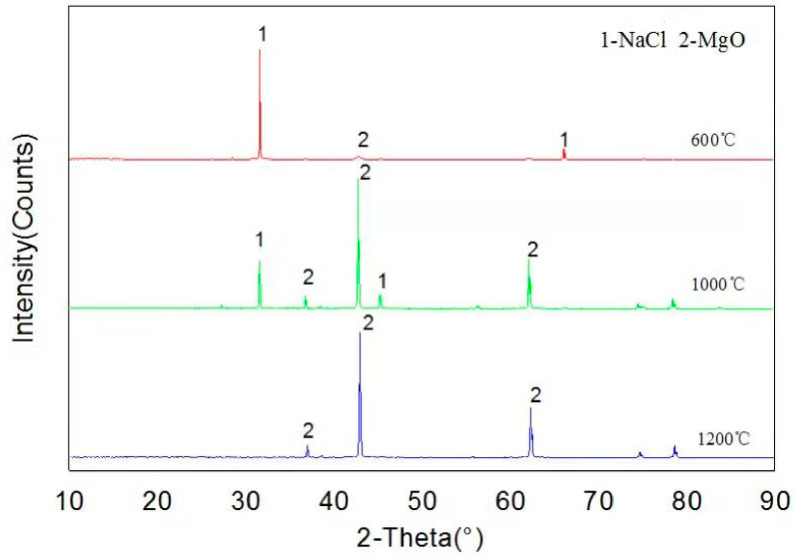
XRD pattern of the reaction products of NaCl and MgO.

As can be seen from Figure 5, XRD patterns show that only MgO and NaCl are present in the samples at temperatures of 600, 1000 and 1200 °C, indicating that MgO has not been corroded by NaCl, and it has good corrosion resistance, which is consistent with thermodynamic calculation results.

Figure 5 shows the XRD spectra of samples after NaCl and MgO high temperature corrosion tests at 600, 1000 and 1200 °C. The results present that only MgO and NaCl were detected in the sample, and no new substance was formed. The experimental results and simulation results by FactSage^TM^ 7.2 software (see Table 3) indicated that MgO is an excellent refractory, which can avoid NaCl corrosion. According to our previous thermodynamic experimental research [23], the thermodynamic model of the software can accurately predict the thermodynamic mechanism of alkali metal corrosion to refractory materials.

## 5. Conclusions

In this paper, different refractory components (MgO·Al_2_O_3_, MgO·Cr_2_O_3_, Al_2_O_3_, Cr_2_O_3_ and MgO) are corroded by different sodium salts (NaCl, Na_2_CO_3_ and Na_2_SO_4_) at different temperatures (600–1200 °C). Thermodynamics studies have obtained the effect of sodium salt type and temperature on the corrosion of refractory materials. By comparison and analysis, the refractory materials with good corrosion resistance are obtained.

1. The temperature has a great influence on the corrosion of refractory materials by sodium salt at 600–1200 °C. Besides MgO and MgO·Al_2_O_3_, the higher the temperature, the stronger the corrosion of refractory materials by sodium sulfate and sodium chloride.
2. Among the five refractory materials, MgO has the best resistance to sodium salt corrosion, followed by MgO·Cr_2_O_3_ and MgO·Al_2_O_3_; Cr_2_O_3_ and Al_2_O_3_ have the worst resistance to sodium salt corrosion. Due to the presence of O_2_, Cr^3+^ is oxidized to Cr^6+^ during corrosion.
3. The accuracy of the thermodynamic calculation of FactSage^TM^ software was verified for the MgO-NaCl system by analyzing the results of thermodynamic experiments. Combined with the author’s previous research work, high-content MgO refractories can solve the corrosion problem of refractories caused by NaCl and KCl.

## Figures and Tables

**Table 1:** Predicted corrosion products and reaction equations of Na_2_CO_3_ on refractory materials at 600–1200 °C.

Species	Temperature (°C)			
	600	700	800	900–1200
Al_2_O_3_	0.16667 mol Na_2_Al_12_O_19_	2 mol NaAlO_2_		
	Equation (1)	Equation (2)		
MgO	-			
Cr_2_O_3_	1 mol Na_2_CrO_4_			
	Equation (4)			
MgO·Cr_2_O_3_	1 mol Na_2_CrO_4_, 0.5mol MgO			
	Equation (5)			
MgO·Al_2_O_3_	0.00962 mol NaAlO_2_, 0.00481 mol MgO	0.08292 mol NaAlO_2_, 0.04146 mol MgO	0.53843 mol NaAlO_2_, 0.26922 mol MgO	2 mol NaAlO_2_, 1 mol MgO
	Equation (3)	Equation (3)	Equation (3)	Equation (3)

“-”: The amount of corrosion products is less than 10^−4^ mol.

**Table 2:** Predicted corrosion products and reaction equations of Na_2_SO_4_ on refractory materials at 600–1200 °C.

Species	Temperature (°C)			
	600–900	1000	1100	1200
Al_2_O_3_	-	0.0003 mol NaAl_9_O_14_	0.0033mol NaAl_9_O_14_	0.031672 mol NaAl_9_O_14_
		Equation (6)	Equation (6)	Equation (6)
MgO	-			
Cr_2_O_3_	-	0.0002 mol Na_2_CrO_4_	0.0013 mol Na _2_CrO_4_	0.0073 mol Na _2_CrO_4_
		Equation (7)	Equation (7)	Equation (7)
MgO·Cr_2_O_3_	-		0.0001 mol Na_2_CrO_4_	0.0008 mol Na_2_CrO_4_, 0.0004mol MgO
			Equation (8)	Equation (8)
MgO·Al_2_O_3_	-			

“-”: The amount of corrosion products is less than 10^−4^ mol.

**Table 3:** Predicted corrosion products and reaction equations of NaCl on refractory materials at 600–1200 °C.

Species	Temperature (°C)				
	600–800	900	1000	1100	1200
Al_2_O_3_	-	0.0001 mol NaAl_9_O_14_	0.0003 mol NaAl_9_O_14_	0.0008 mol NaAl_9_O_14_	0.0019 mol NaAl_9_O_14_
		Equation (9)	Equation (9)	Equation (9)	Equation (9)
MgO		-			
Cr_2_O_3_	-	0.0001 mol Na_2_CrO_4_	0.0002 mol Na_2_CrO_4_	0.0003 mol Na_2_CrO_4_	0.0006 mol Na_2_CrO_4_
		Equation (10)	Equation (10)	Equation (10)	Equation (10)
MgO·Cr_2_O_3_		-			0.0001 mol Na_2_CrO_4_
					Equation (11)
MgO·Al_2_O_3_	-				

“-”: The amount of corrosion products is less than 10^−4^ mol.

## References

[1] Prigent P., Bouchetou M.L., Poirier J. (2011). Andalusite: An amazing refractory raw material with excellent corrosion resistance to sodium vapours. Ceram. Int..

[2] Xu T.T., Xu Y.B., Li Y.W., Sang S.B., Wang Q.H., Zhu T.B., Nath M., Zhang B. (2019). Corrosion mechanisms of magnesia-chrome refractories in copper slag and concurrent formation of hexavalent chromium. J. Alloy. Compd..

[3] Hirata T., Morimoto T., Ohta S., Uchida N. (2003). Improvement of the corrosion resistance of alumina-chromia ceramic materials in molten slag. J. Eur Ceram. Soc..

[4] Bouchetou M.L., Poirier J., Arbelaez Morales L., Chotard T., Joubert O., Weissenbacher M. (2019). Synthesis of an innovative zirconia-mullite raw material sintered from andalusite and zircon precursors and an evaluation of its corrosion and thermal shock performance. Ceram. Int..

[5] Wang X.H., Zhao P.D., Chen J.W., Zhao H.Z., He K. (2018). Corrosion resistance of Al–Cr-slag containing chromium–corundum refractories to slags with different basicity. Ceram. Int..

[6] Hirata T., Akiyama K., Yamamoto H. (2001). Corrosion resistance of Cr_2_O_3_-Al_2_O_3_ ceramics by molten sodium sulphate-vanadium pentoxide. J. Mater. Sci..

[7] Mahnicka-Goremikina L., Svinka R., Svinka V. (2018). Influence of ZrO_2_ and WO_3_ doping additives on the thermal properties of porous mullite ceramics. Ceram. Int..

[8] Kujur M.K., Roy I., Kumar K., Chintaiah P., Ghosh S., Ghosh N.K. (2018). Influence of ZrO_2_ and WO_3_ doping additives on the thermal properties of porous mullite ceramics. Mater. Today: Proc..

[9] Wiedemeier H., Singh M. (1991). Thermal stability of refractory materials for high-temperature composite applications. J. Mater. Sci..

[10] He L.P., Chen D.C., Shang S.P. (2004). Fabrication and wear properties of Al_2_O_3_-SiC ceramic coatings using aluminum phosphate as binder. J. Mater. Sci..

[11] Yoshikawa M., Iida E., Shikama H., Inoue K. (2005). Applicaton of chrome-free bricks for incinerated-ash melting furnaces. J. Tech. Assoc Refract..

[12] Moda J., Tanaka K., Kitamura S. (2008). Chrome-free castables for waste melting furnances. J. Tech. Assoc Refract..

[13] Fan R.D., Zhao H.Z., Zhang H., Zhao P.D., Chen J.W., Wang X.H. (2019). Effect of partial substitution of alumina-chromium slag for Al_2_O_3_ on microstructures and properties of Al_2_O_3_-SiC-C trough castables. Ceram. Int..

[14] Igabo K., Sakida S., Benino Y. (2008). Development of Cr-free refractories for high temperature municipal waste incinerators. J. Tech. Assoc Refract..

[15] Gehre P., Aneziris C.G., Veres D., Parr C., Fryda H., Neuroth M. (2013). Improved spinel-containing refractory castables for slagging gasifiers. J. Eur Ceram. Soc..

[16] Ren B., Li Y.W., Nath M., Wang Q.H., Xu Y.B. (2018). Enhanced alkali vapor attack resistance of bauxite-SiC refractories for the working lining of cement rotary kilns via incorporation of andalusite. Ceram. Int..

[17] Bianco R., Jacobson N. (1989). Corrosion of cordierite ceramics by sodium sulphate at 1000 °C. J. Mater. Sci..

[18] Stjernberg J., Olivas-Ogaz M.A., Antti M.L., Ion J.C., Lindblom B. (2013). Laboratory scale study of the degradation of mullite /corundum refractories by reaction with alkali-doped deposit materials. Ceram. Int..

[19] Boris R., Antonovič V., Keriene J., Stonys R., Kudžma A., Zdanevičius P. (2017). Study of alkali resistance of refractory materials used in boilers operating on wood fuel. Refract. Ind Ceram..

[20] Gregurek D., Schmidl J., Reinharter K., Reiter V., Spanring A. (2018). Copper anode furnace: Chemical, mineralogical and thermo-chemical considerations of refractory wear mechanisms. J. Miner. Metals Mater. Soc..

[21] Stjernberg J., Lindblom B., Wikström J., Antti M.L., Odénc M. (2010). Microstructural characterization of alkali metal mediated high temperature reactions in mullite based refractories. Ceram. Int..

[22] Huang R., Qian X., Lv X.D., Liu P.S., Zhang J.Z. (2018). Slag–refractory interactions during ilmenite smelting: Thermodynamic simulation and experimental data. Refract. Ind Ceram..

[23] Zhao Y., Cheng G.S., Xiang Y., Long F., Dong C.Q. (2018). Thermodynamic study of the corrosion of refractories by sodium carbonate. Materials.

[24] Zhang Z.J. (2006). Physical Chemistry of Materials.

